# Photocontrol of Foam Stability Using Photoresponsive Cationic Surfactants

**DOI:** 10.1021/acs.langmuir.6c03506

**Published:** 2026-10-20

**Authors:** Shiho Yada, Kensuke Kiyohara, Kazuki Yamaguchi, Yukishige Kondo

**Affiliations:** † Department of Industrial Chemistry, Faculty of Engineering, 26413 Tokyo University of Science, 6-3-1 Niijuku, Katsushika-ku, Tokyo 125-8585, Japan; ‡ Research Institute for Science and Technology, Tokyo University of Science, 2641 Yamazaki, Noda, Chiba 278-8510, Japan

## Abstract

Photoresponsive surfactants provide an effective approach for controlling interfacial phenomena with light. In this study, we investigated the light-driven modulation of foam stability using a series of photoresponsive cationic surfactants containing an azobenzene moiety, C_*m*_AzoC_*n*_TAB (*m*/*n* = 2/2, 2/4, 2/6, 4/2, and 6/2). *Trans–cis* photoisomerization under UV irradiation was confirmed by UV–vis and ^1^H NMR analyses. Surface tension measurements revealed that the critical micelle concentration (CMC) increased upon *trans–cis* photoisomerization and decreased with increasing alkyl chain length. Foam stability was evaluated both above CMC and at concentrations corresponding to the maximum surface tension difference between the *trans* and *cis* isomers. Foam stability depended strongly on the terminal alkyl chain length of the hydrophobic tail. Notably, *trans*-C_4_AzoC_2_TAB formed highly stable foams, whereas UV irradiation markedly decreased foam stability via *trans–cis* photoisomerization. Localized UV irradiation further demonstrated that foam collapse occurred selectively in the irradiated region, enabling spatially controlled defoaming. Based on these results, photoisomerization may alter the interfacial adsorption state and generate interfacial nonequilibrium, which may contribute to foam-film destabilization and subsequent foam collapse. These findings demonstrate that photoresponsive surfactants with appropriately designed alkyl chain lengths enable effective, localized, light-driven control of foam stability.

## Graphical abstract

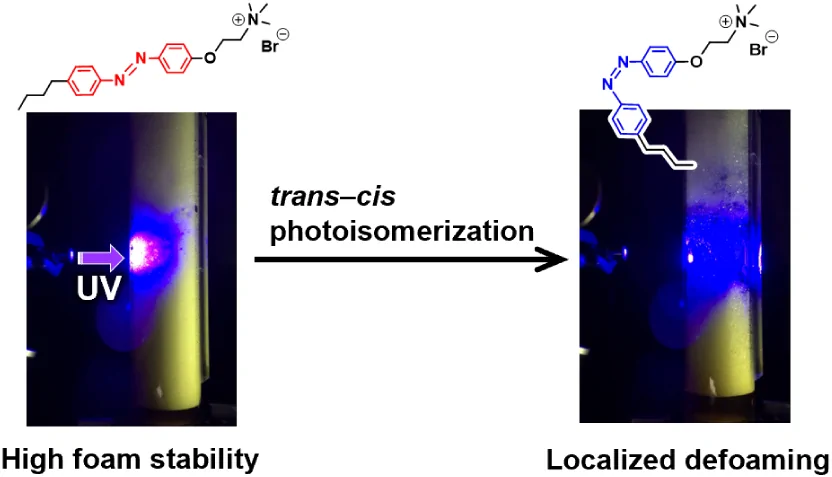

## Introduction

The interfacial properties of surfactant solutions can be controlled by external stimuli such as pH changes,^1,2^ CO_2_,^3^ redox reactions,^4^ and magnetic fields.^5,6^ Accordingly, numerous studies on stimuli-responsive surfactants have been reported.^7,8^ Among these, photoresponsive surfactants containing an azobenzene moiety undergo *trans*-to-*cis* isomerization upon ultraviolet (UV) irradiation, whereas the reverse *cis*-to-*trans* isomerization occurs under visible-light irradiation or thermal treatment. Thus, their interfacial properties can be reversibly switched by irradiation at specific wavelengths. Owing to this unique feature, photoresponsive surfactants have been widely studied for controlling interfacial properties,^9,10^ the structure of molecular assemblies,^11–13^ emulsion stability,^14–16^ viscosity,^17^ and directional droplet motion.^18^

Foams are dispersions of gas bubbles in water that are stabilized by amphiphilic molecules adsorbed at the gas–liquid interface. Foams play important roles in many industrial processes; however, in some applications their suppression or rapid collapse is required.^19^ Therefore, controlling foam stability is an important practical issue. Bubble lifetime depends strongly on the properties of surfactants in the aqueous phase. Surfactants not only reduce surface tension and facilitate bubble formation but also play a key role in stabilizing the foam films at the interface. Foam stability is governed by multiple factors, including electrostatic repulsion between bubbles and the Gibbs–Marangoni effect arising from surface tension gradients at the interface.^20^ Conventionally, defoaming agents have been used to control foams; however, limitations such as short-lived efficacy, contamination of the system, and the need for additional treatment have been noted.^21^ As a result, reversible control of foam stability using external stimuli has attracted increasing attention. In recent years, foam control using stimuli-responsive surfactants has been reported in response to pH,^22,23^ redox reactions,^24^ and temperature.^25^ Light is particularly attractive because it offers high temporal and spatial control, enabling localized manipulation of foams. Nevertheless, only a limited number of studies have applied photoresponsive surfactants to foam control.^26–32^ Lei et al.^26^ demonstrated photoresponsive foams using a rigid surfactant derived from dehydroabietic acid, while Braunschweig et al.^27,28^ investigated light-responsive air–water interfaces and photoresponsive aqueous foams using arylazopyrazole-based surfactants. Salonen et al. reported light- and temperature-responsive emulsion foams.^29^ Although these studies demonstrated the feasibility of controlling foam behavior using photoresponsive surfactants, they mainly focused on demonstrating photoresponsiveness of specific surfactant systems. To the best of our knowledge, the influence of surfactant molecular structure, particularly alkyl chain length, on foam stability and light-induced defoaming behavior has not been systematically investigated. In addition, the molecular structure of surfactants—especially alkyl chain length—strongly influences intermolecular interactions and interfacial adsorption and is therefore a key determinant of foam stability.

In this study, we investigated light-driven control of foam stability using photoresponsive cationic surfactants containing an azobenzene moiety, C_*m*_AzoC_*n*_TAB (*m*/*n* = 2/2, 2/4, 2/6, 4/2, and 6/2; Figure 1). These molecular structures were selected to examine the effects of the two alkyl chain lengths (*m* and *n*) on the interfacial properties and light-responsive foam behavior. Specifically, one alkyl chain length was fixed at two carbon atoms while the other was varied, with C_2_AzoC_2_TAB serving as the reference structure. This molecular design allows the influence of each alkyl chain length to be examined separately within the investigated series. Therefore, this study aims to clarify how variations in alkyl chain length influence the interfacial properties, foam stability, and light-responsive foam behavior of the investigated C_*m*_AzoC_*n*_TAB surfactants.

**Figure 1:**
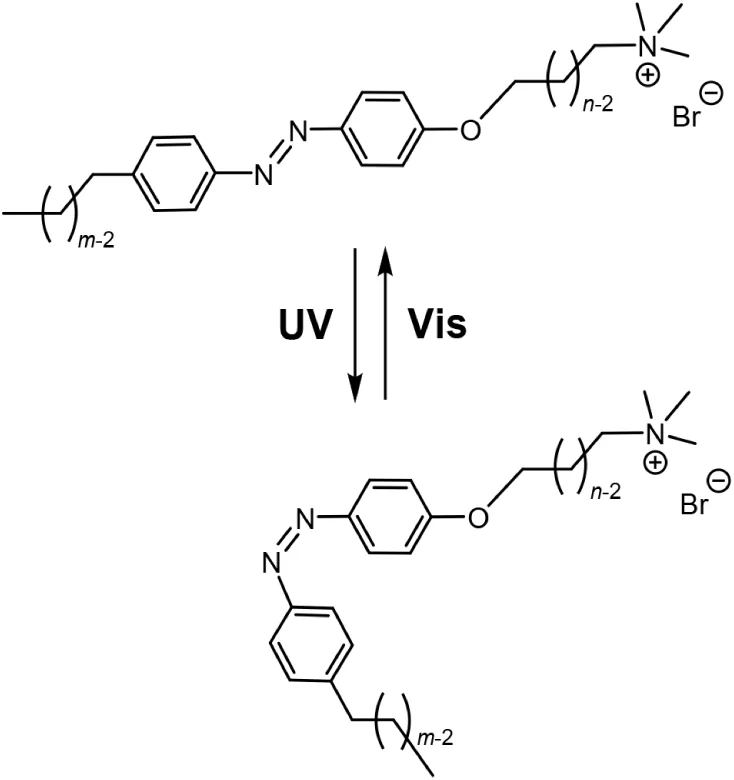
*Trans–cis* photoisomerization of **C*_m_* AzoC*_n_* TAB**.

## Experimental Section

### Materials

The photoresponsive surfactant C_*m*_AzoC_*n*_TAB was synthesized via a Williamson reaction of 4-(4-alkylphenylazo)­phenol with 1,2-dibromoalkane, followed by quaternization with trimethylamine in methanol, according to a previously reported method.^33^ Detailed procedures, ^1^H NMR data, ESI-MS data, and elemental analysis results for C_*m*_AzoC_*n*_TAB are provided in the Supporting Information.

The *trans*–*cis* isomerization of the azobenzene-based compounds was induced by UV irradiation. Hereafter, we designate the photostationary state of C_*m*_AzoC_*n*_TAB aqueous solutions stored under laboratory conditions (ambient light and temperature) as the “*trans* state” or “*trans* form.” Similarly, we refer to samples obtained after UV irradiation (λ = 300–400 nm) as the “*cis* state” or “*cis* form.”

### Measurements

Unless otherwise specified, aqueous C_*m*_AzoC_*n*_TAB solutions used in the following measurements were prepared using Milli-Q Plus water (resistivity = 18.2 MΩ·cm).

### Determination of the *Trans*-to-*Cis* Photoisomerization Ratio under UV Irradiation

The time required to reach the photostationary state upon UV irradiation of an aqueous solution of *trans*-C_*m*_AzoC_*n*_TAB was monitored by UV–vis spectroscopy (V-750, JASCO Corp., Tokyo, Japan). UV irradiation was performed using a Handy UV Lamp (LUV-16; 365 nm) positioned 6 cm from the vial, providing an irradiance of 0.38 μW cm^–2^.

The fractions of the *trans* and *cis* isomers before and after UV irradiation were quantitatively determined by ^1^H NMR spectroscopy (BioSpin AVANCE NEO 400, Bruker). ^1^H NMR spectra were acquired before and after irradiating various solutions with UV light (Handy UV Lamp, LUV-16; 365 nm) for 10 min, with the lamp positioned 4 cm from the NMR tube (0.86 μW cm^–2^). The *cis*-isomer fraction before and after irradiation was calculated from the integral values of the azobenzene proton signals, and the *trans*-to-*cis* photoisomerization ratio was determined. For these measurements, the surfactant concentration was set to 0.5 mmol dm^–3^, below the critical micelle concentration (CMC) because micellization often causes broadening and chemical shifts of the NMR signals. Hereafter, the samples before UV irradiation and those at the photostationary state after UV irradiation are referred to as the *trans* and *cis* states, respectively.

### Equilibrium Surface Tension and Electrical Conductivity

The surface tensions of the C_*m*_AzoC_*n*_TAB surfactants in aqueous solution were measured for both the *trans* and *cis* states in the dark by the pendant drop method (DM300, Kyowa Interface Science Co., Ltd., Saitama, Japan). Droplets of surfactant solution were generated from a needle dispenser (0.7 mm diameter). Optical images of the droplet were acquired with a monochrome CCD camera equipped with a white LED (Kyowa Interface Science Co., Ltd.), and the images were analyzed using FAMAS software (Kyowa Interface Science Co., Ltd.). For surface tension measurements of the *cis* state, solutions were first irradiated with UV light (Handy UV Lamp, LUV-16; 365 nm) for 30 min, with the lamp positioned 4 cm from the vial (0.38 μW cm^–2^). The droplet was then measured while being irradiated with UV light using the LAX-Cute equipped with a UV-A (300–400 nm) mirror module, as reported previously.^9^ During the measurement, the UV lamp of the LAX-Cute was positioned 5 cm from the measurement cell at an angle of 45° relative to the illumination axis of the white-light source, providing a UV irradiance of 22.4 μW cm^–2^. Data were recorded under white-light illumination for photographic observation. Although visible light can induce *cis–trans* photoisomerization of azobenzene derivatives, no appreciable change in the photoisomerization state was detected under the specific white-light illumination conditions used in the present measurement system, as confirmed by the nearly unchanged UV–vis absorption spectra before and after exposure to the white-light illumination.^9^

The electrical conductivities of the C_*m*_AzoC_*n*_TAB surfactants in aqueous solution were measured for both the *trans* and *cis* states in the dark using a multiparameter water quality meter (MM-43X, DKK-TOA Corp., Tokyo, Japan). For measurements of the cis state, the solutions were irradiated under the same conditions described above prior to the conductivity measurements. The CMC was determined from the intersection of the linear regions below and above the breakpoint in the conductivity–concentration plots.

Measurements were performed at 25.0 ± 0.5 °C.

### Foam Stability

Foam stability was evaluated in the dark using a dynamic foam analyzer (DFA100, KRÜSS, Germany). A *trans*-C_*m*_AzoC_*n*_TAB surfactant solution (25 mL) was poured into a glass cylinder, and air was introduced from the bottom at 0.5 L min^–1^ to generate foam. When the foam reached a height of 200 mm, air injection was stopped. Foam height was monitored according to a previously reported procedure.^34^ Using the same setup, the generated foam was irradiated with UV light using the LAX-Cute equipped with a UV-A (300–400 nm) mirror module positioned 10 cm above the top of the foam (5.6 μW cm^–2^). Foam height was measured for 1 h at 1 min intervals, and the results obtained with and without UV irradiation were compared. To examine the temporal evolution of bubble morphology, bubble images were recorded at 5 min intervals using the imaging system of the dynamic foam analyzer at a fixed height of 100 mm above the bottom of the glass cylinder during the foam-stability measurements.

For localized foam-control experiments, foam generated in the DFA100 cell was irradiated locally at the center of the foam with UV light from a distance of 8 cm (8.8 μW cm^–2^), and the time-dependent changes were recorded.

### Time-Dependent Surface Tension under UV Irradiation

To examine time-dependent changes in the interfacial properties of C_4_AzoC_2_TAB upon UV irradiation, the surface tension of an aqueous *trans*-C_4_AzoC_2_TAB solution (1.1 mmol dm^–3^) was measured by the Wilhelmy plate method using a platinum plate and a tensiometer (tensiio1, KRÜSS, Germany) at room temperature. The surface tension was first monitored without UV irradiation, after which the sample was irradiated with UV light using the LAX-Cute equipped with a UV-A (300–400 nm) mirror module positioned 4.5 cm from the sample (27.7 μW cm^–2^), while the surface tension measurement was continued.

### Dynamic Surface Tension

The dynamic surface tensions of aqueous C_*m*_AzoC_*n*_TAB solutions for both the *trans* and *cis* states were measured by the maximum bubble pressure method using a dynamic surface tension analyzer (BP100, KRÜSS, Germany). Measurements were performed at the respective *C*_Δγmax_ concentration for each surfactant, where *C*_Δγmax_ denotes the concentration at which the difference in equilibrium surface tension between the *trans* and *cis* states reached its maximum (Δγ_max_). For measurements of the *cis* state, the solutions were irradiated with UV light for 30 min using a Handy UV Lamp (LUV-16; 365 nm) positioned 4 cm from the sample prior to the measurements (0.9 μW cm^–2^). All measurements were performed in the dark at 25.0 ± 0.5 °C, and dynamic surface tension was recorded as a function of surface age for both the *trans* and *cis* states.

### Reversibility of Photoisomerization

The reversibility of the *trans*–*cis* photoisomerization was examined using an aqueous *trans*-C_4_AzoC_2_TAB solution (0.5 mmol dm^–3^). The solution was alternately irradiated with UV light (Handy UV Lamp, LUV-16; 365 nm) and visible light (LAX-Cute; 400–700 nm) for 5 min each. During UV irradiation, the lamp was positioned 6 cm from the vial, providing an irradiance of 0.38 μW cm^–2^. During visible-light irradiation, the lamp was positioned 5 cm from the vial, providing an irradiance of 37.4 μW cm^–2^.

## Results and Discussion

### Determination of the Photoisomerization Ratio

First, UV–vis spectra were recorded during UV irradiation of *trans*-C_*m*_AzoC_*n*_TAB to monitor *trans–cis* photoisomerization of the azobenzene moiety. Figure 2a shows the UV–vis spectra of an aqueous solution of *trans*-C_4_AzoC_2_TAB (0.5 mmol dm^–3^) recorded during 10 min of UV irradiation. Figure S1 shows the time-dependent changes in the absorption spectra of aqueous *trans*-C_*m*_AzoC_*n*_TAB solutions (0.5 mmol dm^–3^) during 10 min of UV irradiation. Before irradiation, a peak at ∼352 nm was observed and assigned to the π–π* transition of the azobenzene moiety.^35^ Upon UV irradiation, a characteristic signal assigned to the n−π* transition of the *cis* isomer appeared at ∼440 nm. Because the π–π* absorption of the *trans* isomer is much stronger than the n−π* absorption of the *cis* isomer, the extent of photoisomerization was evaluated by monitoring the decrease in the *trans* absorption during UV irradiation. Figure 2b shows the time-dependent change in the relative absorbance at 352 nm for aqueous C_*m*_AzoC_*n*_TAB solutions (0.5 mmol dm^–3^). For all C_*m*_AzoC_*n*_TAB derivatives, the absorbance reached a constant value after ∼420 s of UV irradiation, indicating attainment of the photostationary state. These results suggest that the photoisomerization rate is not markedly affected by alkyl chain length (*m* and *n*).

**Figure 2:**
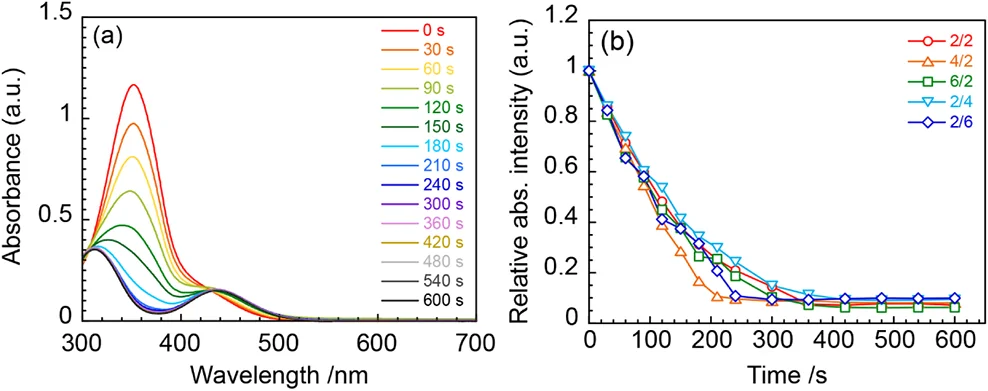
(a) Time evolution of the UV–vis absorption spectra of an aqueous C_4_AzoC_2_TAB solution (0.5 mmol dm^–3^) during UV irradiation at different irradiation times from 0 to 600 s. (b) Time evolution of the relative absorbance at 352 nm of aqueous C*_m_* AzoC*_n_* TAB solutions (0.5 mmol dm^–3^) during UV irradiation, normalized to the absorbance at 0 s.

Next, we quantified the photoisomerization ratio of C_*m*_AzoC_*n*_TAB in D_2_O (0.5 mmol dm^–3^) by ^1^H NMR. Figure 3 shows the ^1^H NMR spectra of C_4_AzoC_2_TAB before UV irradiation (*trans* isomer) and after 10 min of UV irradiation (*cis* isomer). Prior to irradiation, the observed signals were predominantly assigned to the *trans* isomer. After 10 min of UV irradiation, however, most signals corresponded to the *cis* isomer, indicating efficient *trans–cis* photoisomerization. The *trans*/*cis* molar ratios were estimated from the integrated intensities of the aromatic proton signals in the ^1^H NMR spectra. The *trans*:*cis* ratio of C_4_AzoC_2_TAB was determined to be 93:7 before UV irradiation and 3:97 after 10 min of UV irradiation. Figure S2 shows the ^1^H NMR spectra of C_*m*_AzoC_*n*_TAB with different alkyl chain lengths (*m*, *n*). The same analysis revealed that, for all C_*m*_AzoC_*n*_TAB derivatives, the *trans* fraction before UV irradiation exceeded ∼92%, whereas the *cis* fraction after UV irradiation exceeded ∼96%. These results demonstrate efficient trans–cis photoisomerization of C_*m*_AzoC_*n*_TAB in aqueous solution under UV irradiation.

**Figure 3:**
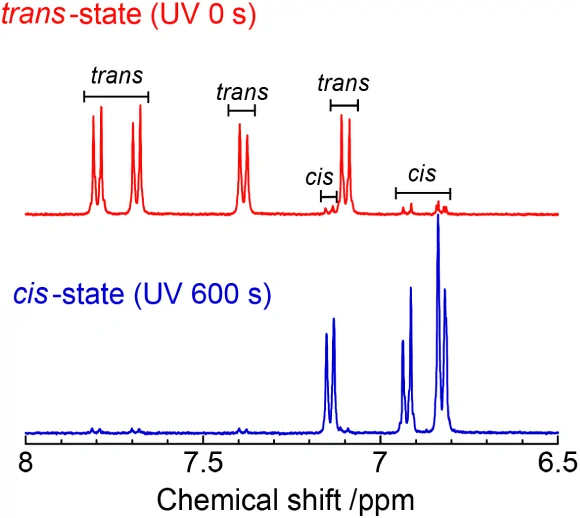
^1^H NMR spectra of *trans*- and *cis*-C_4_AzoC_2_TAB in D_2_O (0.5 mmol dm^–3^). The *trans* state is the photostationary state under ambient light, and the cis state is obtained after UV irradiation for 600 s.

### Surface Tension

Figure 4a shows surface tension plots for the photoresponsive cationic surfactants C_*m*_AzoC_*n*_TAB. Figure 4b shows the differences between the surface tension plots of the *trans* and *cis* states (Δγ = γ_*cis*_ – γ_*trans*_) as a function of concentration. Figure S3 shows the conductivity plots as a function of surfactant concentration. The CMC values determined from the surface tension and conductivity measurements, together with the surface tension at the CMC (γ_CMC_), are summarized in Table 1.

**Table 1:** CMC and γ_CMC_ Values of C*_m_* AzoC*_n_* TAB at 25 °C

*m*/*n*		CMC^a^/mmol dm^–3^	CMC^b^/mmol dm^–3^	γ_CMC_/mN m^–1^
2/2	*trans*	9.84 ± 1.46	9.2	45.5 ± 0.1
	*cis*	12.9 ± 0.9	12.0	47.1 ± 0.1
2/4	*trans*	4.05 ± 0.21	4.4	44.0 ± 0.1
	*cis*	7.74 ± 0.43	8.1	41.9 ± 0.1
2/6	*trans*	1.78 ± 0.03	1.8	46.0 ± 0.2
	*cis*	3.26 ± 0.07	2.2	45.5 ± 0.2
4/2	*trans*	3.15 ± 0.07	2.5	40.0 ± 0.2
	*cis*	5.34 ± 0.09	4.7	40.0 ± 0.2
6/2	*trans*	0.70 ± 0.02	0.57	38.6 ± 0.1
	*cis*	1.07 ± 0.01	0.71	38.6 ± 0.1

a Determined from surface tension measurements.

b Determined from conductivity measurements. CMC, critical micelle concentration; γ_CMC_, surface tension at CMC.

**Figure 4:**
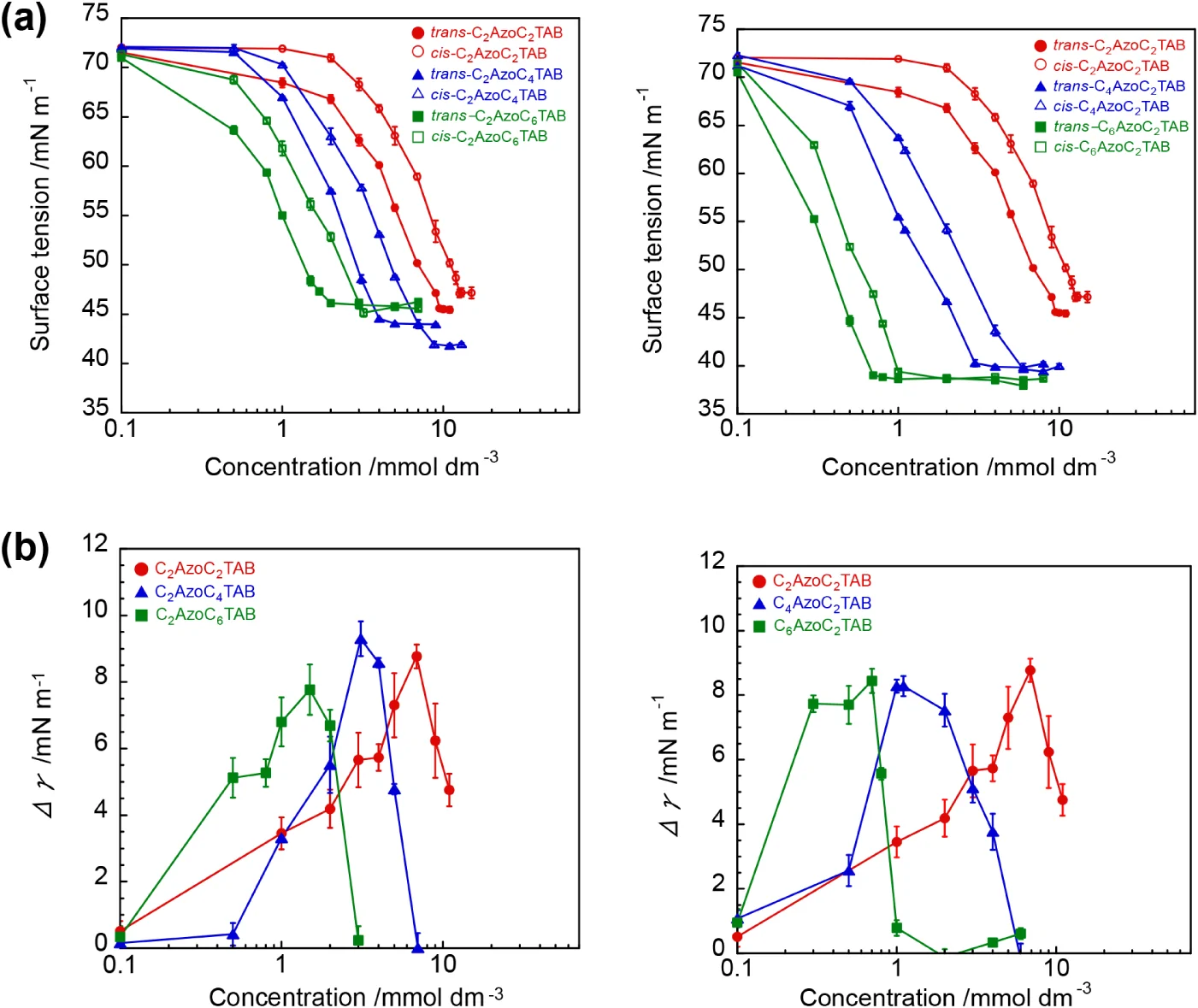
(a) Relationships between surface tension and concentration for C*_m_* AzoC*_n_* TAB. Error bars represent standard deviations of three independent measurements (*n* = 3). (b) Difference in surface tension between the *trans* and *cis* states (Δγ = γ_*cis*_ – γ*_trans_*).

In all systems, the CMC obtained from surface tension measurements was higher for the *cis* isomer than for the *trans* isomer. Symmetrical *trans*-azobenzene is nonpolar, whereas bent *cis*-azobenzene is polar because of a large dipole moment across the azo linkage.^36–38^ These results suggest that micelle formation is less favorable for the *cis* isomer than for the *trans* isomer, leading to a higher CMC for the *cis* isomer. In addition, CMC decreased with increasing alkyl chain lengths *m* and *n*. These CMC trends are consistent with those reported for azobenzene-based surfactants by Hayashita et al.^39^ In general, the relationship between the logarithm of the CMC and the alkyl chain length *n* is described by the Klevens equation:^40^

1 log(CMC)=A−Bn

where *A* and *B* are constants yielding a linear correlation. From the plots of log­(CMC) versus alkyl chain lengths *m* and *n* (Figure S4), the *B* values for *trans*-C_*m*_AzoC_2_TAB, *cis*-C_*m*_AzoC_2_TAB, *trans*-C_2_AzoC_*n*_TAB, and *cis*-C_2_AzoC_*n*_TAB were determined to be 0.28, 0.27, 0.18, and 0.15, respectively.

Compared with typical ionic surfactants, which generally exhibit *B* values of approximately 0.3,^41^ the *B* values for C_2_AzoC_*n*_TAB (0.15–0.18) are reduced by about half. This reduction suggests that a bulky azobenzene moiety near the terminal region of the hydrophobic group suppresses micelle formation.

The surface tension at CMC (γ_CMC_) was approximately 42–47 mN m^–1^ for the *m*/*n* = 2/2, 2/4, and 2/6 surfactants, whereas lower values of approximately 39–40 mN m^–1^ were obtained for the *m*/*n* = 4/2 and 6/2 surfactants. These results suggest that the terminal alkyl chain length (*m*) plays an important role in determining the surface-tension-lowering ability of the investigated C_*m*_AzoC_*n*_TAB surfactants. The adsorption behavior and orientation of C_*m*_AzoC_*n*_TAB at the air/water interface have not yet been fully clarified; therefore, the interfacial orientation will be investigated in detail in future studies using X-ray and neutron reflectivity.

Based on the surface tension measurements, foam stability was evaluated at concentrations below the CMC where the surface tension difference reached a maximum (Δγ_max_), namely the concentration at Δγ_max_ (*C*_Δγmax_). For C_*m*_AzoC_*n*_TAB (*m*/*n* = 2/2, 2/4, 2/6, 4/2, and 6/2), the Δγ_max_ values were 8.8, 9.3, 7.8, 8.3, and 8.4 mN m^–1^, respectively, and the corresponding *C*_Δγmax_ values were 6.9, 3.1, 1.5, 1.1, and 0.7 mmol dm^–3^, respectively. In addition, to assess foam stability at high concentration, the same experiments were performed at a fixed concentration of 10 mmol dm^–3^.

### Influence of UV Light Irradiation on C*_m_* AzoC*_n_* TAB Foam Stability

Figure 5 shows the time dependence of the relative foam height, normalized to the initial foam height immediately after foaming, for aqueous solutions of *trans*-C_*m*_AzoC_*n*_TAB at *C*_Δγmax_ with and without UV irradiation. The relationship between the relative foam height after 30 min (*H*_30 min_) and molecular structure (*m*/*n*) is summarized in Figure 6a. For *trans*-C_2_AzoC_*n*_TAB, foam stability was low for all investigated alkyl chain lengths *n*, even without UV irradiation; accordingly, although UV irradiation promoted foam collapse, the overall effect was relatively small. In contrast, the alkyl chain length *m* of *trans*-C_*m*_AzoC_2_TAB strongly affected foam stability. *trans*-C_*m*_AzoC_2_TAB with *m* ≥ 4 exhibited high relative foam heights, with *H*_30 min_ exceeding 90%. Upon UV irradiation, *H*_30 min_ decreased to 9.7% and 85% for *m* = 4 and 6, respectively, indicating that the effect of UV irradiation on foam stability depended strongly on the alkyl chain length *m*.

**Figure 5:**
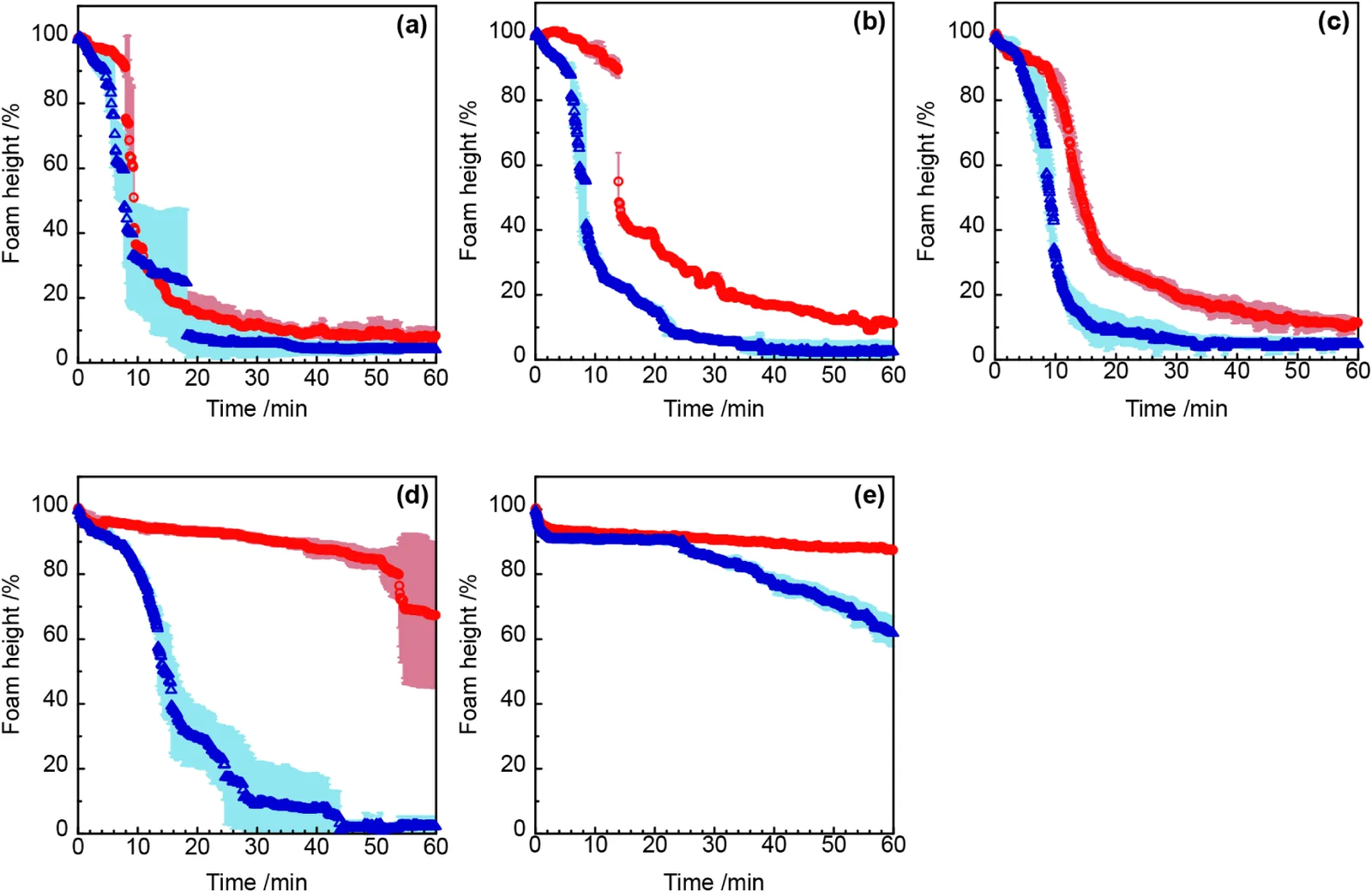
Relationships between foam height and time for *trans*-**C*_m_* AzoC*_n_* TAB** at *C*_Δγmax_: (a) C_2_AzoC_2_TAB, (b) C_2_AzoC_4_TAB, (c) C_2_AzoC_6_TAB, (d) C_4_AzoC_2_TAB, (e) C_6_AzoC_2_TAB. Red: kept in the dark; blue: irradiated from above with UV. Error bars represent standard deviations of three independent measurements (*n* = 3).

**Figure 6:**
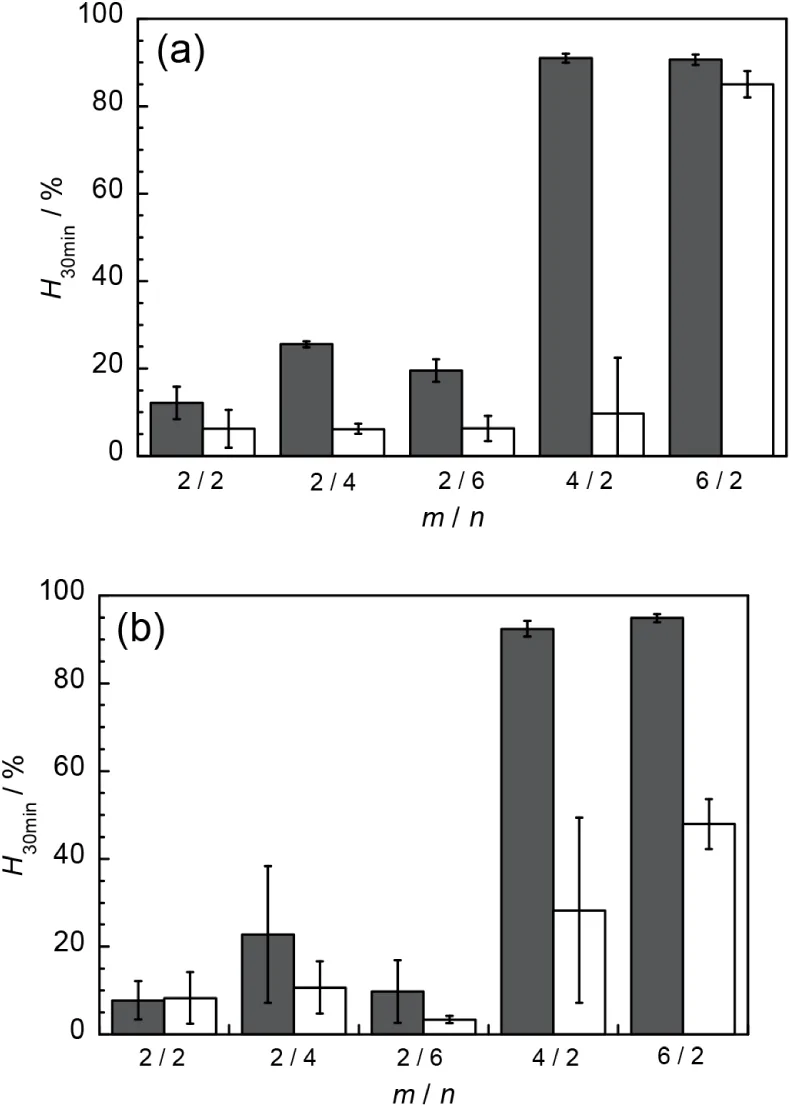
Relationship between foam stability (*H*_30 min_) and the molecular structure (*m*/*n*) of *trans*-C*_m_* AzoC*_n_* TAB, kept in the dark (gray) and exposed to UV from the top (white). (a) *C*_Δγmax_. (b) 10 mmol dm^–3^. Error bars represent standard deviations of three independent measurements (*n* = 3).

Figure S5 shows the time dependence of the relative foam height, normalized to the initial foam height immediately after foaming, for aqueous solutions of *trans*-C_*m*_AzoC_*n*_TAB at 10 mmol dm^–3^ (above the CMC) with and without UV irradiation. The relationship between the relative foam height after 30 min (*H*_30 min_) and molecular structure (*m*/*n*) is summarized in Figure 6b. Similar to the results obtained at *C*_Δγmax_, *trans*-C_2_AzoC_*n*_TAB exhibited low foam stability for all alkyl chain lengths *n*, even without UV irradiation; therefore, although UV irradiation promoted foam collapse, the overall effect was relatively small. In contrast, the alkyl chain length *m* of *trans*-C_*m*_AzoC_2_TAB strongly influenced foam stability. Without UV irradiation, foams formed by *trans*-C_*m*_AzoC_2_TAB were highly stable, with *H*_30 min_ exceeding 90% for *m* ≥ 4. Upon UV irradiation, *H*_30 min_ decreased to 28% and 48% for *m* = 4 and 6, respectively.

These results demonstrate that C_4_AzoC_2_TAB enables effective UV-triggered control of foam stability over a wide concentration range, both below CMC (1.1 mmol dm^–3^) and above CMC (10 mmol dm^–3^).

To further characterize the foam destabilization process beyond the changes in foam height, the temporal evolution of bubble morphology was examined using images obtained with the dynamic foam analyzer. Figures S6 and S7 show the bubble morphology of foams formed from aqueous solutions of *trans-*C_m_AzoC_n_TAB at *C*_Δγmax_ and 10 mmol dm^–3^, respectively, with and without UV irradiation. The images were recorded at a fixed height of 100 mm above the bottom of the foam column, while the initial foam height was 200 mm. At the initial stage, the foams formed at 10 mmol dm^–3^ generally appeared to consist of finer bubbles than those formed at *C*_Δγmax_. In contrast, no simple systematic relationship between alkyl chain length and bubble size was apparent from the images. Thus, the pronounced dependence of foam stability on the molecular structure of C*_m_* AzoC*_n_* TAB cannot be explained solely by differences in the initial bubble size and is likely also related to differences in the interfacial properties of the surfactants, as discussed below. The temporal evolution of the bubble morphology also varied depending on the molecular structure and irradiation conditions. Because the images were acquired at a single fixed height within the foam column, these observations reflect the local bubble behavior at this position and do not necessarily represent morphological changes throughout the entire foam.

Localized foam control was examined using C_4_AzoC_2_TAB, which enables effective foam control over a wide concentration range. Only the center of the foam (prepared as described above) was irradiated with UV light, and the time-dependent changes were recorded. The results are shown in Figure 7. Upon UV irradiation, defoaming occurred only in the irradiated region, and complete foam collapse was observed approximately 45 s after irradiation began. These results indicate that C_4_AzoC_2_TAB enables spatially localized foam control by targeted light irradiation. A video of the experimental observations is provided as Movie S1.

**Figure 7:**
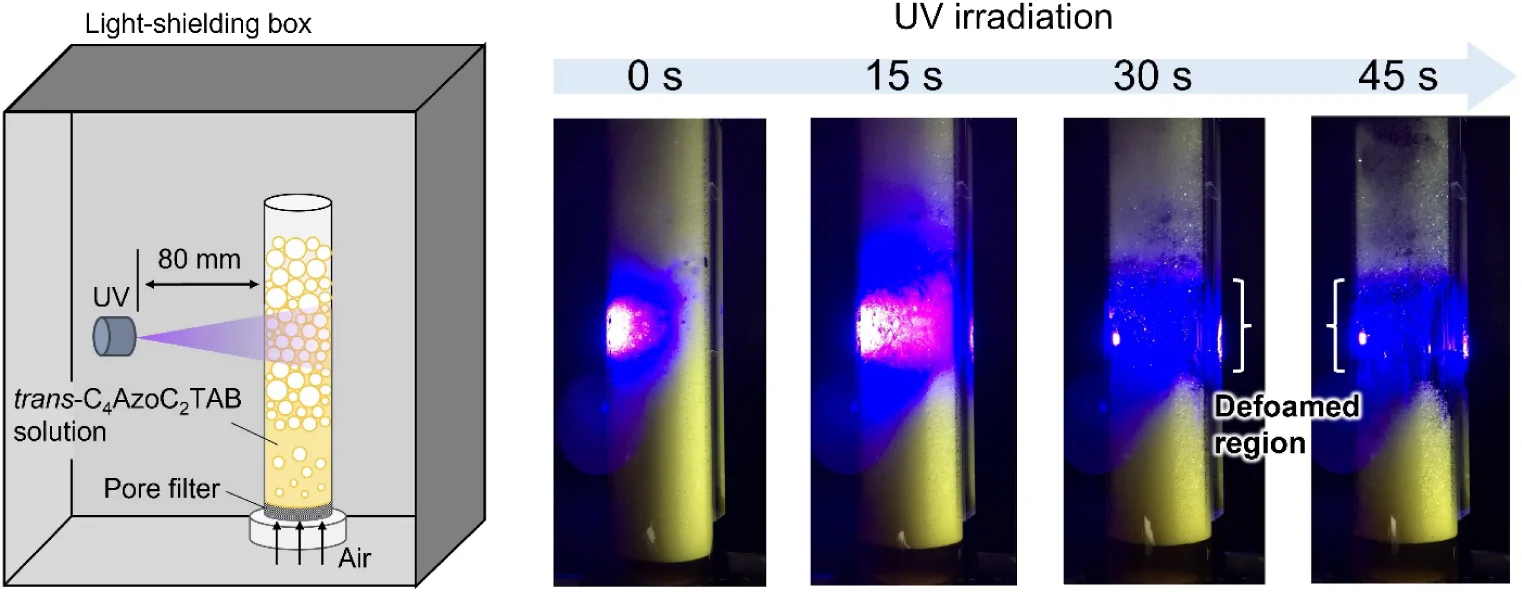
Schematic illustration of the experimental setup and photographs of foam formed from a *trans*-C_4_AzoC_2_TAB solution at 10 mmol dm^–3^ during localized UV light irradiation. The elapsed time after the onset of UV irradiation is indicated above each photograph.

Notably, the time scale of localized foam collapse (approximately 45 s) was substantially shorter than the approximately 420 s required to approach the photostationary state in the UV–vis measurements. Reaching the photostationary state, however, is not necessarily required for foam destabilization. Partial photoisomerization during UV irradiation may already alter the interfacial adsorption state and generate interfacial nonequilibrium within the foam films; therefore, macroscopic foam collapse may occur before the photostationary state is reached. However, because the degree of photoisomerization at the foam interface during localized UV irradiation was not directly determined in the present study, the quantitative relationship between interfacial photoisomerization kinetics and foam-collapse kinetics remains to be established.

### Possible Mechanisms of Defoaming by UV Light Irradiation

This section discusses the origin of the marked decrease in foam stability of an aqueous *trans*-C_4_AzoC_2_TAB solution upon UV irradiation. As described above, the equilibrium surface tension of C_4_AzoC_2_TAB decreases with increasing concentration for both the *trans* and *cis* isomers and reaches a constant value above a certain concentration. The γ_CMC_ values are nearly identical for the *trans* and *cis* isomers, suggesting that the equilibrium surface tension is not the direct cause of the defoaming observed upon UV irradiation. The magnitude of the photoinduced change in equilibrium surface tension observed for the present C_*m*_AzoC_*n*_TAB surfactants is moderate compared with that reported for some previously studied photoresponsive surfactants. For example, Shang et al. reported differences in saturated surface tension of up to 14.4 mN m^–1^ for azobenzene-based nonionic surfactants, whereas Honnigfort et al. reported a substantially larger surface tension change (Δγ = 27 mN m^–1^) for an ionic arylazopyrazole-based surfactant.^28,42^ In the latter system, the large surface tension response was associated with pronounced photoinduced reorganization of the interfacial layer, including a monolayer-to-bilayer transition. These comparisons suggest that the pronounced change in foam stability observed in the present system may be associated with dynamic interfacial changes during trans–cis photoisomerization rather than with the magnitude of the equilibrium surface tension change itself. Theodoratou et al. reported that semifluorinated alkyl azobenzene derivatives form an ordered monolayer in the trans state, whereas photoisomerization to the cis form results in a randomly packed structure at the air/water interface.^43^ This finding demonstrates that trans–cis photoisomerization can substantially alter interfacial molecular organization. Although the molecular orientation and intermolecular interactions of C_4_AzoC_2_TAB at the interface were not directly characterized in the present study, analogous photoinduced changes in interfacial organization may contribute to the observed change in foam stability.

Upon UV irradiation, the azobenzene moieties of C_*m*_AzoC_*n*_TAB in the foam undergo *trans–cis* photoisomerization. To experimentally examine the associated change in the interfacial state, the surface tension of a *trans*-C_4_AzoC_2_TAB solution (1.1 mmol dm^–3^) was monitored before and during UV irradiation (Figure 8). Upon UV irradiation, the surface tension rapidly increased from approximately 49 mN m^–1^ and eventually reached approximately 54–55 mN m^–1^. This increase indicates a photoinduced change in the interfacial properties of C_4_AzoC_2_TAB. In their theoretical study, Chevallier et al. reported that the desorption rate constant of the *cis* isomer of C_4_AzoC_3_TAB at the air/water interface is 2 orders of magnitude higher than that of the *trans* isomer.^32^ Given the structural similarity between C_4_AzoC_3_TAB and C_4_AzoC_2_TAB, enhanced desorption following *trans–cis* photoisomerization may also contribute to the UV-induced increase in surface tension observed for C_4_AzoC_2_TAB. However, because the present surface tension measurements do not directly identify the molecular species leaving the interface, preferential desorption of the *cis* isomer cannot be conclusively established from the present data alone. In general, when surfactant isomerization generates a surface tension gradient, surfactant molecules migrate from regions of low surface tension to regions of high surface tension via the Gibbs–Marangoni effect, thereby stabilizing the foam film.^44^ However, the upper region of the foam formed in the glass cell is far from the bulk aqueous phase. Consequently, readsorption of surfactant molecules from the bulk solution to the foam interface may not be sufficiently rapid to compensate for photoinduced changes in interfacial adsorption. Such photoinduced changes in interfacial adsorption may generate interfacial nonequilibrium within the foam films, potentially including local surface tension gradients. This interfacial nonequilibrium may contribute to destabilization of the foam films and subsequent foam collapse. In addition to the Gibbs–Marangoni effect, photoisomerization may also alter the electrostatic disjoining pressure within the foam films. Because *trans–cis* photoisomerization may alter the adsorption state and local surface concentration of the cationic surfactant, the electrostatic interactions between adjacent bubble surfaces may also be modified during UV irradiation. Although the electrostatic disjoining pressure was not directly measured in the present study, changes in this contribution may act together with the interfacial nonequilibrium to destabilize the foam films and contribute to foam collapse.

**Figure 8:**
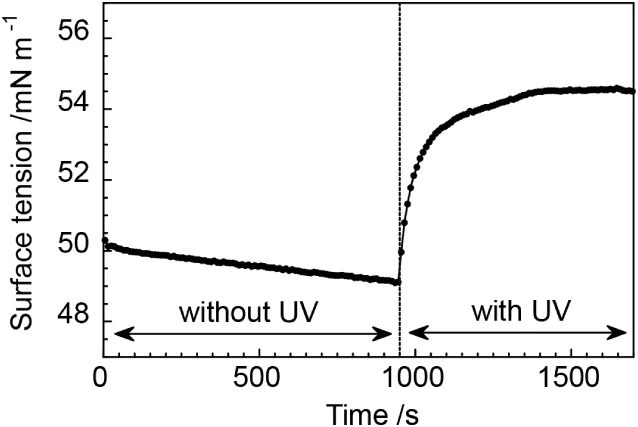
Time evolution of the surface tension of an aqueous C_4_AzoC_2_TAB solution (1.1 mmol dm^–3^), initially in the trans form, before and during UV irradiation at room temperature. The vertical dotted line indicates the onset of UV irradiation.

Next, we consider the effect of the alkyl chain lengths (*m*, *n*) of C_*m*_AzoC_*n*_TAB on foam stability. Hardt et al. investigated the adsorption and orientation at the air/water interface of photoresponsive surfactants containing an arylazopyrazole (AAP) moiety, which is structurally similar to azobenzene.^45^ They reported that introducing long terminal alkyl chains on AAP enhances lateral intermolecular interactions at the air/water interface, yielding a more densely packed molecular arrangement than that of surfactants without such alkyl chains. By analogy, the dependence of foam stability on the terminal alkyl chain length (*m*) observed for C*_m_* AzoC*_n_* TAB may be related to differences in lateral intermolecular interactions at the air/water interface. A short terminal alkyl chain (*m* = 2) may provide weaker lateral interactions, whereas longer chains (*m* = 4 and 6) could enhance such interactions and thereby contribute to the higher foam stability observed experimentally. The dynamic surface tension measurements at the respective *C*_Δγmax_ concentrations revealed that the time-dependent interfacial responses of the investigated C*_m_* AzoC*_n_* TAB surfactants differed between the *trans* and *cis* states and depended on molecular structure (Figure S8). In particular, C_4_AzoC_2_TAB showed a clear difference between the *trans* and *cis* dynamic surface tension profiles over the measured surface-age range, whereas for C_6_AzoC_2_TAB, the two profiles differed at intermediate surface ages but approached similar surface tension values at longer surface ages. These results indicate that *trans–cis* isomerization affects the time-dependent interfacial response in a molecular-structure-dependent manner. Such differences in dynamic interfacial behavior may partly contribute to the different photoresponsive foam behaviors observed among the surfactants. Among the C*_m_* AzoC*_n_* TAB surfactants examined in this study, C_4_AzoC_2_TAB exhibited a particularly pronounced photoresponsive change in foam stability. Direct structural characterization of the interfacial organization of C*_m_* AzoC*_n_* TAB, for example, by X-ray or neutron reflectivity, will be necessary to verify these proposed molecular-level interpretations.

Repeated *trans–cis* photoswitching of C_4_AzoC_2_TAB was further examined by UV–vis spectroscopy (Figure 9). Changes in the absorption band associated with the π–π* transition were observed over five alternating irradiation cycles, indicating that repeated photoswitching was maintained under the present conditions. However, the absorption intensity did not fully return to its initial value after visible-light irradiation, indicating incomplete recovery of the initial *trans*-rich state. Similar incomplete recovery after visible-light irradiation has been reported for other azobenzene-based surfactants.^39^ Although the reversibility of the macroscopic foam response over repeated irradiation cycles was not examined in the present study, the observed molecular photoswitching suggests the potential for reversible light-controlled modulation of foam stability.

**Figure 9:**
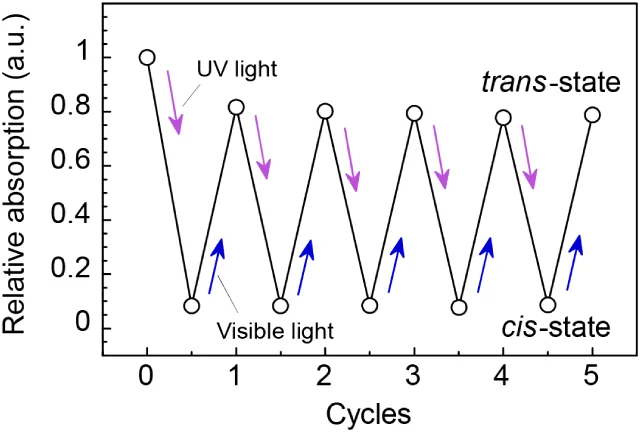
Cycles of the UV–vis absorption intensity corresponding to the π–π* transition of the azobenzene group for C_4_AzoC_2_TAB at 0.5 mmol dm^–3^. During the cycle, the sample solution was alternately irradiated with UV light (λ = 365 nm) and visible light (λ = 400–700 nm).

## Conclusion

In this study, we investigated the light-induced control of foam stability using azobenzene-based, photoresponsive cationic surfactants (C_*m*_AzoC_*n*_TAB) with different alkyl chain lengths. Spectroscopic measurements confirmed *trans–cis* photoisomerization and indicated that the isomerization kinetics were not markedly affected by alkyl chain length. Surface tension measurements showed that CMC increased upon *trans–cis* photoisomerization and decreased with increasing alkyl chain length. Foam-stability measurements indicated a strong dependence on surfactant molecular structure. Among the surfactants investigated in this study, *trans*-C_4_AzoC_2_TAB exhibited high foam stability and a particularly pronounced decrease in foam stability upon UV irradiation via *trans–cis* photoisomerization. Localized UV irradiation experiments demonstrated that defoaming occurred selectively in the irradiated region, indicating that light can induce spatially controlled foam collapse. These results suggest that photoinduced changes in interfacial adsorption upon *trans–cis* isomerization may generate interfacial nonequilibrium within the foam films, thereby contributing to foam-film destabilization and subsequent foam collapse. Overall, this study demonstrates that the molecular structure of azobenzene-based photoresponsive surfactants influences their light-responsive foam behavior and that, among the surfactants investigated here, C_4_AzoC_2_TAB enables localized light-driven control of foam stability.

Although the present study demonstrates the feasibility of light-driven foam control using azobenzene-based photoresponsive surfactants, several challenges remain for practical applications. In particular, the penetration of UV light may be limited in opaque or highly scattering foams, and the long-term durability of repeated photoisomerization cycles as well as the environmental compatibility of azobenzene-based surfactants should be further evaluated. In addition, because foam stability can be affected by environmental conditions such as temperature and ionic strength, the robustness of the present system under different temperatures and salt concentrations should be systematically evaluated. Further systematic investigation of a broader range of molecular structures, including additional combinations of *m* and *n*, will also be necessary to establish more general structure–property relationships. Future optimization of the molecular design and irradiation conditions, together with evaluation under practically relevant conditions, will be important for extending this approach to practical foam-control systems.

## Supplementary Material





## Notes

The synthesis procedures, ^1^H NMR, ESI-MS, and elemental analysis data of *trans*-C_*m*_AzoC_*n*_TAB, together with UV–vis absorption spectra, ^1^H NMR spectra, conductivity data, the relationship between the CMC and alkyl chain length, relationships between foam height and time, images of the foam structure, and dynamic surface tension data of *trans*- and *cis*-C_*m*_AzoC_*n*_TAB (PDF)
Video of the experimental observations (MP4)
