# Supplementary material for: Photocontrol of Foam Stability Using Photoresponsive Cationic Surfactants
Source: Langmuir. 2026 Oct 20;42(43):31717–26. doi: 10.1021/acs.langmuir.6c03506 (PMC13738745; doi:10.1021/acs.langmuir.6c03506)
Supplement: Supplementary file 1 [file la6c03506_si_001.pdf]

Supporting Information

Photocontrol of Foam Stability Using Photoresponsive Cationic Surfactants

Shiho Yada[1], Kensuke Kiyohara[1], Kazuki Yamaguchi[1], and Yukishige Kondo[1, 2*]*

[1] Department of Industrial Chemistry, Faculty of Engineering, Tokyo University of Science, 6-3-1 Niijuku, Katsushika-ku, Tokyo 125-8585, Japan

[2] Research Institute for Science and Technology, Tokyo University of Science, 2641 Yamazaki, Noda, Chiba 278-8510, Japan

*Corresponding authors. Email: yada@rs.tus.ac.jp (S.Y.) and ykondo@rs.tus.ac.jp (Y.K.)

Contents

S1. Synthesis procedures

S1.1. Materials

S1.2. Synthesis of 4-(4-alkylphenylazo)phenol (C_mAzoOH)

S1.3. Synthesis of 2-(4-(4-alkylphenyl)diazenylphenoxy)-1-bromoalkane (C_mAzoC_nBr)

S1.4. Synthesis of 2-(4-(4-alkylphenyl)diazenylphenoxy)alkyltrimethylammonium bromide (C_mAzoC_nTAB)

S2. ^{1}H NMR, elemental analysis and ESI-MS data

S2.1. ^{1}H NMR

S2.2. Elemental analysis

S2.3. ESI-MS (positive mode)

Figure S1. Time-evolution of the UV–vis absorption spectra of the C_mAzoC_nTAB aqueous solutions during UV-irradiation

Figure S2. ^{1}H NMR spectra of *trans*- and *cis*-C_mAzoC_nTAB in D_2O

Figure S3. Variation in conductivity with surfactant concentration for C_mAzoC_nTAB

Figure S4. Relationship between the CMC and alkyl chain length for C_mAzoC_nTAB

Figure S5. Relationships between foam height and time for *trans*-C_mAzoC_nTAB

Figure S6. Time-dependent images of the foam structure for C_mAzoC_nTAB solution at 10 mmol dm^{-3}

Figure S7. Time-dependent images of the foam structure for C_mAzoC_nTAB solution at $C_{\Delta\gamma max}$

Figure S8. Dynamic surface-tension profiles of the *trans* and *cis* states of C_mAzoC_nTAB surfactants

The Supporting Information includes 8 Figures on 14 pages in total.

S1. Synthesis procedures

S1.1. Materials.

4-Alkylaniline, 1,2-dibromoethane, phenol and trimethylamine (ca. 25% in methanol, ca. 3.2 mol L^{-1}) were purchased from Tokyo Chemical Industry Co., Ltd. (Tokyo, Japan). Acetone, chloroform, chloroform-d1, ethanol, hexane, hydrochloric acid, anhydrous magnesium sulfate, potassium carbonate, sodium carbonate and sodium nitrite were purchased from Kanto Chemical Co., Inc. (Tokyo, Japan). Sodium chloride was purchased from FUJIFILM Wako Pure Chemical Corp. (Osaka, Japan). All chemicals were of reagent grade and were used without further purification.

S1.2. Synthesis of 4-(4-alkylphenylazo)phenol (C_mAzoOH).

4-Alkylaniline (1.0 equiv.) was converted to the corresponding azo compound (C_mAzoOH) via diazotization with sodium nitrite (1.0 equiv.) in aqueous hydrochloric acid at 3 °C, followed by azo coupling with phenol (1.0 equiv.) in the presence of sodium carbonate (3.0 equiv.). The reaction mixture was acidified to pH 3 with concentrated hydrochloric acid and extracted with chloroform. The organic layer was dried over anhydrous sodium sulfate, and the solvent was removed under reduced pressure. Recrystallization from hexane afforded C_mAzoOH as a brown solid.

S1.3. Synthesis of 2-(4-(4-alkylphenyl)diazenylphenoxy)-1-bromoalkane (C_mAzoC_nBr).

C_mAzoOH (1.0 equiv.) and potassium carbonate (4.0 equiv.) were suspended in acetone and stirred at 60 °C for 30 min. 1,2-Dibromoethane (6.0 equiv.) was then added, and the mixture was heated under reflux at 60 °C for 5 h. After cooling to room temperature, the mixture was filtered, and the filtrate was concentrated under reduced pressure. The crude product was recrystallized from ethanol to afford C_mAzoC_nBr as a yellow–orange solid.

S1.4. Synthesis of 2-(4-(4-alkylphenyl)diazenylphenoxy)alkyltrimethylammonium bromide (C_mAzoC$_n$TAB).

C_mAzoC$_n$Br (1.0 equiv.) was reacted with trimethylamine (25% in methanol, 10 equiv.) at 70 °C for 24 h. After cooling to room temperature, the mixture was filtered and the filtrate was concentrated under reduced pressure. The crude product was recrystallized from acetone to afford C_mAzoC$_n$TAB as a yellow–orange solid.

The yields were as follows: C_2AzoC$_2$TAB: 30%, C_2AzoC$_4$TAB: 44 %, C_2AzoC$_6$TAB: 44%, C_4AzoC$_2$TAB: 33%, C_6AzoC$_2$TAB: 29%

S2. ^{1}H NMR, elemental analysis and ESI-MS data

The structure of **C_mAzoC$_n$TAB** was determined by ^{1}H-NMR (Bruker, AVANCE NEO 400 (400 MHz), Billerica, MA, USA), elemental analysis (Perkin-Elmer 2400II CHNS/O, Waltham, MA, USA), and ESI-MS (Sciex X500R QTOF, Tokyo, Japan).

S2.1. ^{1}H NMR:

C_2AzoC$_2$TAB (ppm, solvent $CDCl_3$) : δ 1.29 (3H, t, J = 10.0 Hz), 2.73 (2H, q, J = 12.0 Hz), 3.61 (9H, s), 4.39-4.45 (2H, brs), 4.57-4.64 (2H, brs), 7.05 (2H, d, J = 20.0 Hz), 7.33 (2H, d, J = 16.0 Hz), 7.82 (2H, d, J = 16.0 Hz), 7.93 (2H, d, J = 24.0 Hz).

C_2AzoC$_4$TAB (ppm, solvent $CDCl_3$): δ 1.29 (3H, t, J = 10.0 Hz), 1.91-2.09 (4H, m), 2.73 (2H, q, J = 12.0 Hz), 3.48 (9H, s), 3.84 (2H, t, J = 18.0 Hz), 4.14 (2H, t, J = 16.0 Hz), 7.01 (2H, d, J = 20.0 Hz), 7.33 (2H, d, J = 20.0 Hz), 7.81 (2H, d, J = 24.0 Hz), 7.89 (2H, d, J = 36.0 Hz).

C_2AzoC$_6$TAB (ppm, solvent d_6-acetone) : δ 1.28 (3H, t, *J* = 12.0 Hz), 1.50-1.60 (2H, m), 1.60-1.69 (2H, m), 1.86-1.93 (2H, m), 1.96-2.01 (2H, m), 2.75 (2H, q, *J* = 10.7 Hz), 3.43 (9H, s), 3.71 (2H, t, *J* = 14.0 Hz), 4.16 (2H, t, *J* = 12.0 Hz), 7.08 (2H, d, *J* = 56 Hz), 7.43 (2H, d, *J* = 20 Hz), 7.83 (2H, d, *J* = 16 Hz), 7.92 (2H, d, *J* = 20 Hz).

C_4AzoC$_2$TAB (ppm, solvent d_6-acetone) : δ 0.95 (3H, t, *J* = 10.0 Hz), 1.33-1.44 (2H, m), 1.61-1.70 (2H, m), 2.72 (2H, t, *J* = 12.0 Hz), 3.60 (9H, s), 4.27 (2H, t, *J* = 8.0 Hz), 4.78-4.83 (2H, brs), 7.24 (2H, d, *J* = 20.0 Hz), 7.41 (2H, d, *J* = 20.0 Hz), 7.82 (2H, d, *J* = 16.0 Hz), 7.93(2H, d, *J* = 16.0 Hz).

C_6AzoC$_2$TAB (ppm, solvent d_6-acetone) : δ 0.76 (3H, t, *J* = 14.0 Hz), 1.14-1.30 (6H, m), 1.50-1.60 (2H, m), 2.58 (2H, t, *J* = 16.0 Hz), 3.48 (9H, s), 4.11-4.18 (2H, brs), 4.64-4.71 (2H, brs), 7.12 (2H, d, *J* = 20.0 Hz), 7.29 (2H, d, *J* = 20.0 Hz), 7.70 (2H, d, *J* = 20.0 Hz), 7.81 (2H, d, *J* = 20.0 Hz).

S2.2. Elemental analysis:

C_2AzoC$_2$TAB. Calcd.for $C_{19}H_{26}N_3OBr\ H_2O$: C, 55.61; H, 6.88; N, 10.24. Found: C, 55.18; H, 6.53; N, 10.19.

C_2AzoC$_4$TAB. Calcd.for $C_{21}H_{30}N_3OBr\ 0.5H_2O$: C, 58.74; H, 7.28; N, 9.79. Found: C, 58.94; H, 7.21; N, 9.64.

C_2AzoC$_6$TAB. Calcd.for $C_{23}H_{34}N_3OBr\ H_2O$: C, 59.22; H, 7.78; N, 9.01. Found: C, 59.01; H, 7.49; N, 9.02.

C_4AzoC$_2$TAB. Calcd.for $C_{21}H_{30}N_3OBr$: C, 60.00; H, 7.19; N, 10.00. Found: C, 60.64; H, 7.14; N, 10.01.

C_6AzoC$_2$TAB. Calcd.for $C_{23}H_{34}N_3OBr$: C, 61.60; H, 7.64; N, 9.37. Found: C, 61.48; H, 7.31; N, 9.41.

S2. 3. ESI-MS (positive mode):

C_2AzoC$_2$TAB. Calcd for $[M – Br]^+$: 312.21, Found :312.21.

C_2AzoC$_4$TAB. Calcd for $[M – Br]^+$: 340.24, Found :340.24.

C_2AzoC$_6$TAB. Calcd for $[M – Br]^+$: 368.27, Found :368.27.

C_4AzoC$_2$TAB. Calcd for $[M - Br]^+$: 340.24, Found :340.24.

C_6AzoC$_2$TAB. Calcd for $[M - Br]^+$: 368.27, Found :368.27.

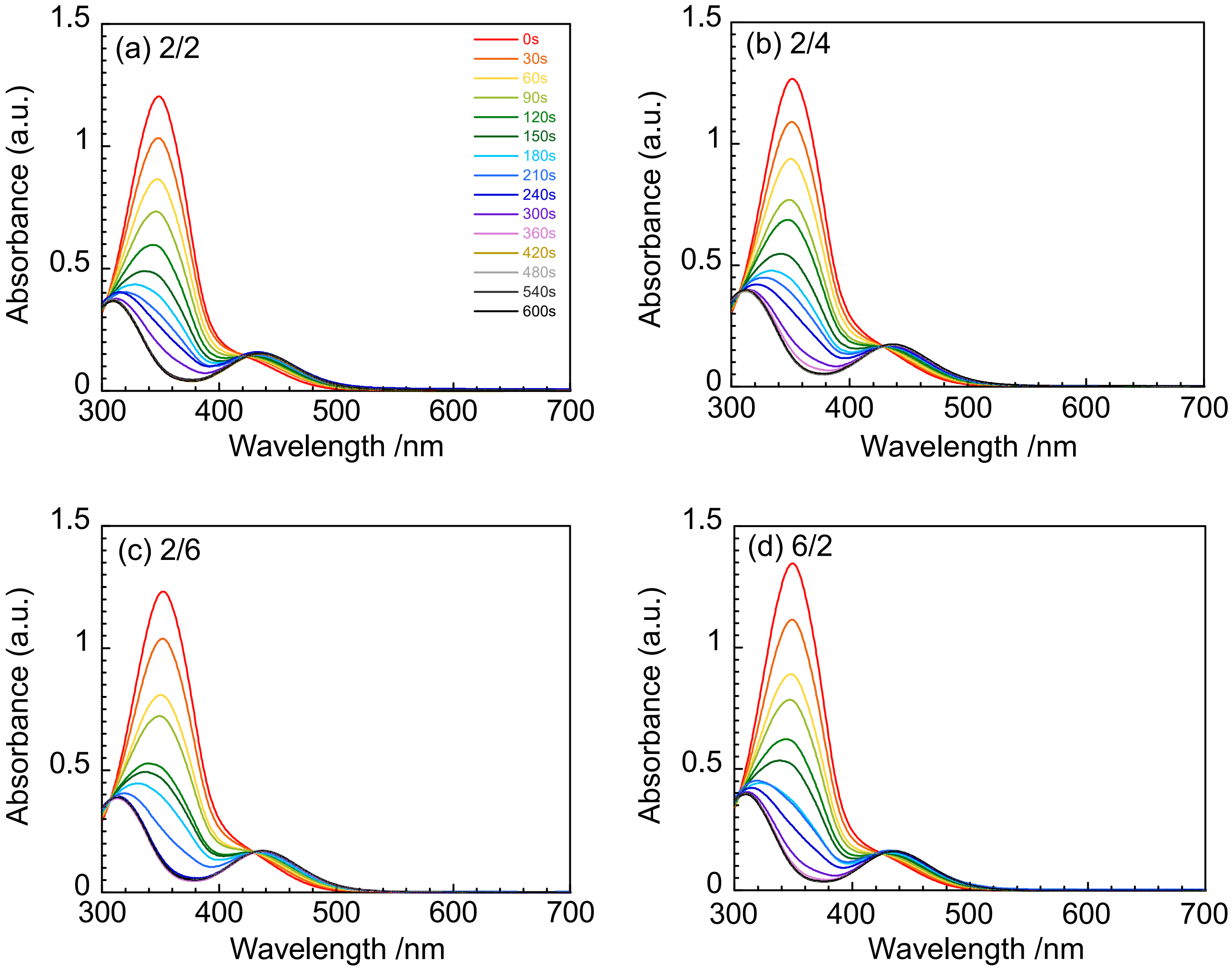

Figure S1. Time-evolution of the UV–vis absorption spectra of the C_mAzoC_nTAB aqueous solutions at 0.5 mmol dm^{-3} during UV-irradiation.; a) C_2AzoC_2TAB, b) C_2AzoC_4TAB, c) C_2AzoC_6TAB, d) C_6AzoC_2TAB.

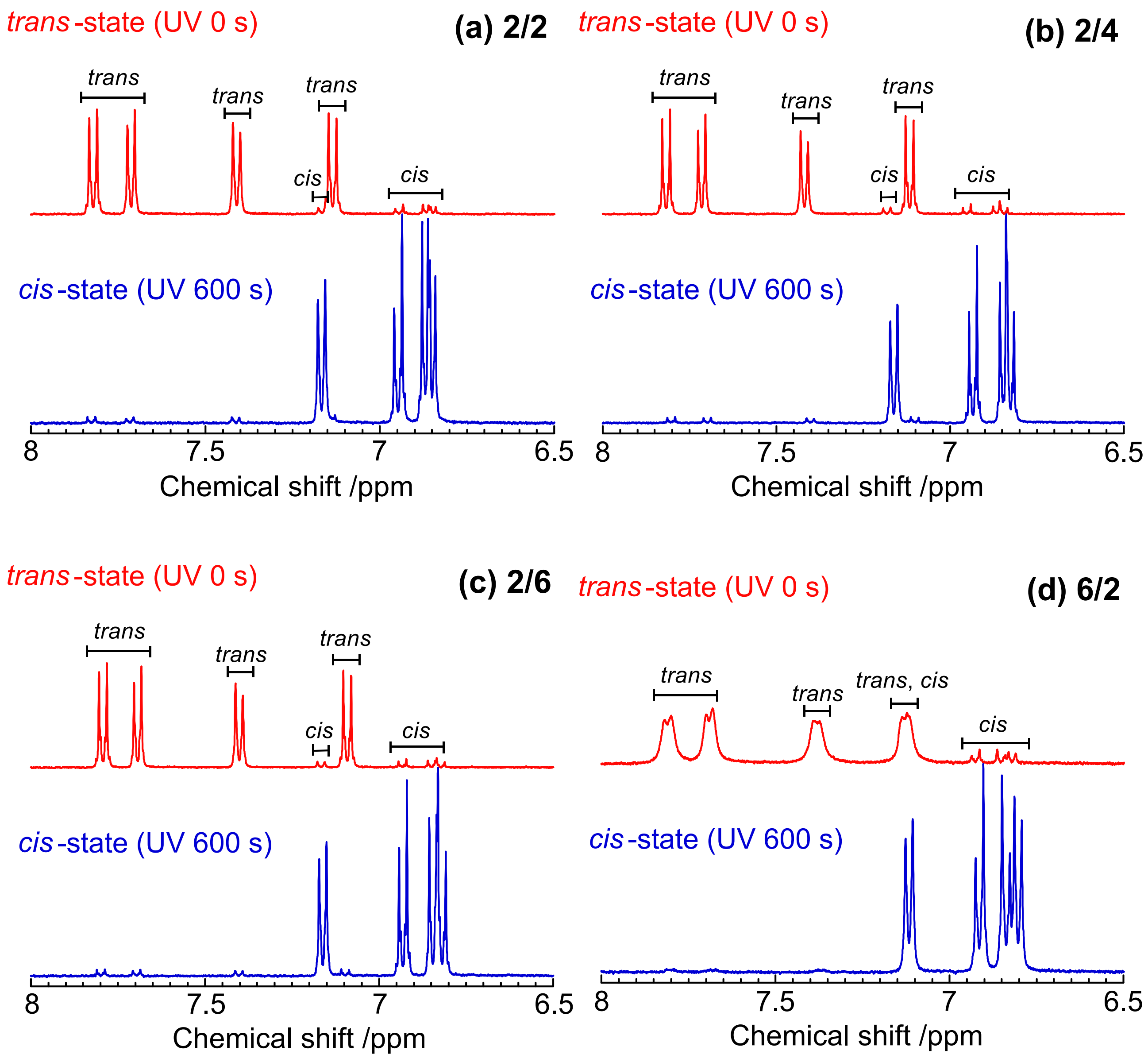

Figure S2. ^{1}H NMR spectra of *trans*- and *cis*-C_mAzoC_nTAB in D_2O at 0.5 mmol dm^{-3}; a) C_2AzoC_2TAB b) C_2AzoC_4TAB c) C_2AzoC_6TAB d) C_6AzoC_2TAB. The *trans* state is the photo-stationary state under the ambient light and the cis state is obtained after the UV-light irradiation for 600 s.

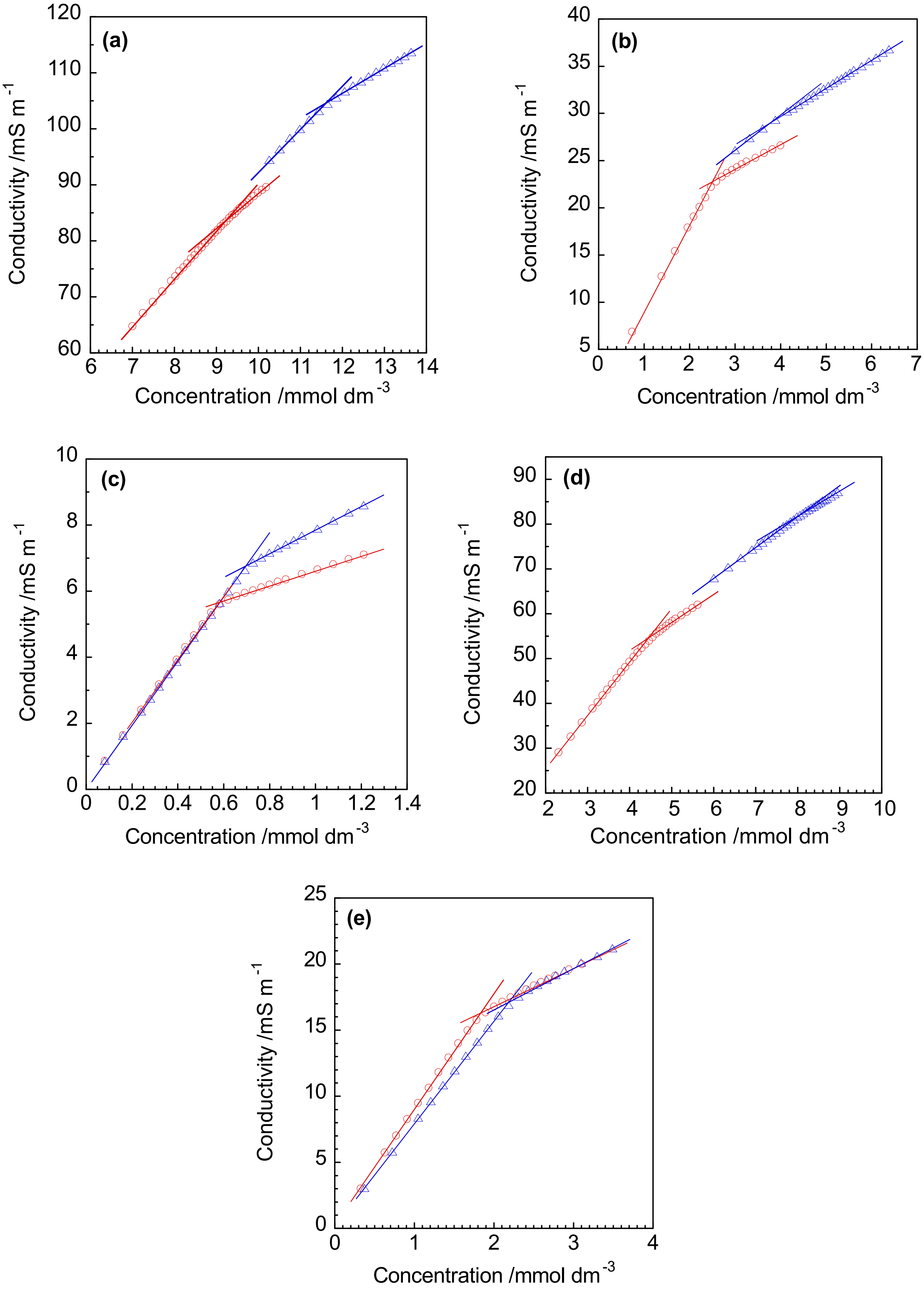

Figure S3. Variation in conductivity with surfactant concentration for C_mAzoC_nTAB: ○ (red open circles), *trans* isomer; △ (blue open squares), *cis* isomer. (a) C_2AzoC_2TAB (b) C_4AzoC_2TAB (c) C_6AzoC_2TAB (d) C_2AzoC_4TAB (e) C_2AzoC_6TAB.

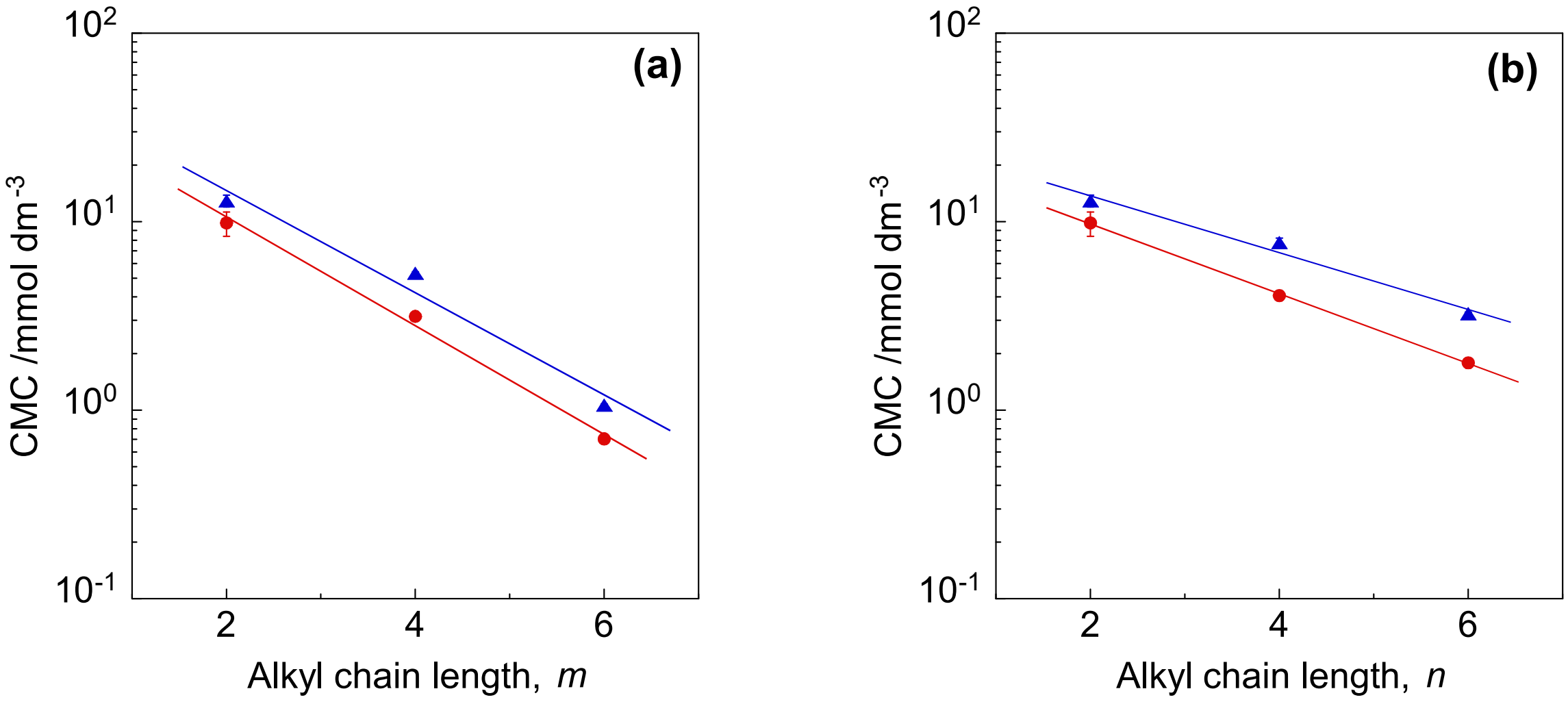

Figure S4. (a) Relationship between the CMC and alkyl chain length for C_mAzoC_2TAB: ●, *trans* isomer; ▲, *cis* isomer. (b) Relationship between the CMC and alkyl chain length for C_2AzoC_nTAB:●, *trans* isomer; ▲, *cis* isomer.

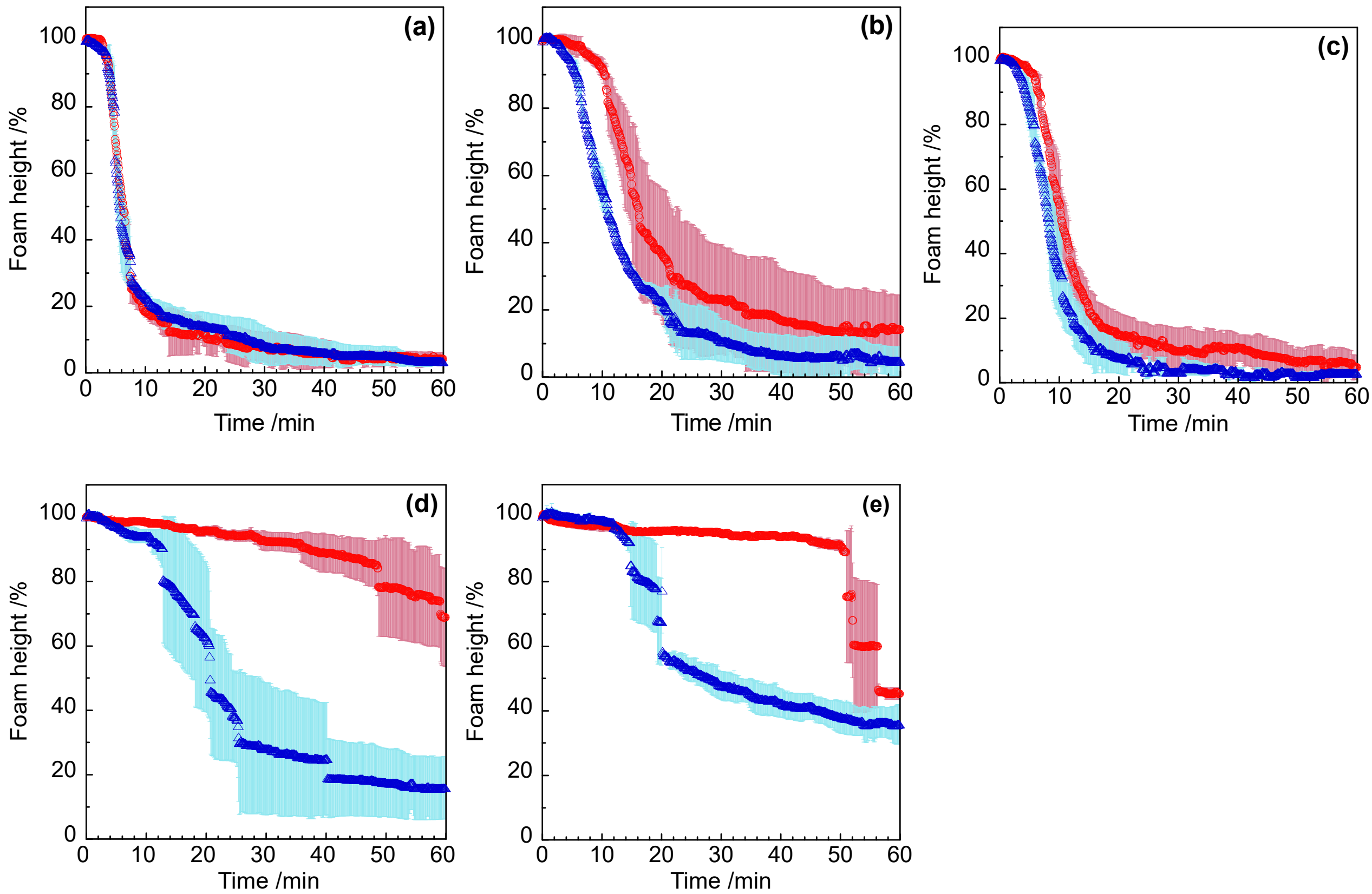

Figure S5. Relationships between foam height and time for *trans*-C_mAzoC_nTAB at 10 mmol dm^{-3}; a) C_2AzoC_2TAB b) C_2AzoC_4TAB c) C_2AzoC_6TAB d) C_4AzoC_2TAB e) C_6AzoC_2TAB. Red: kept in the dark, Blue: exposed from the top to UV. Error bars represent standard deviations of three measurements (n = 3).

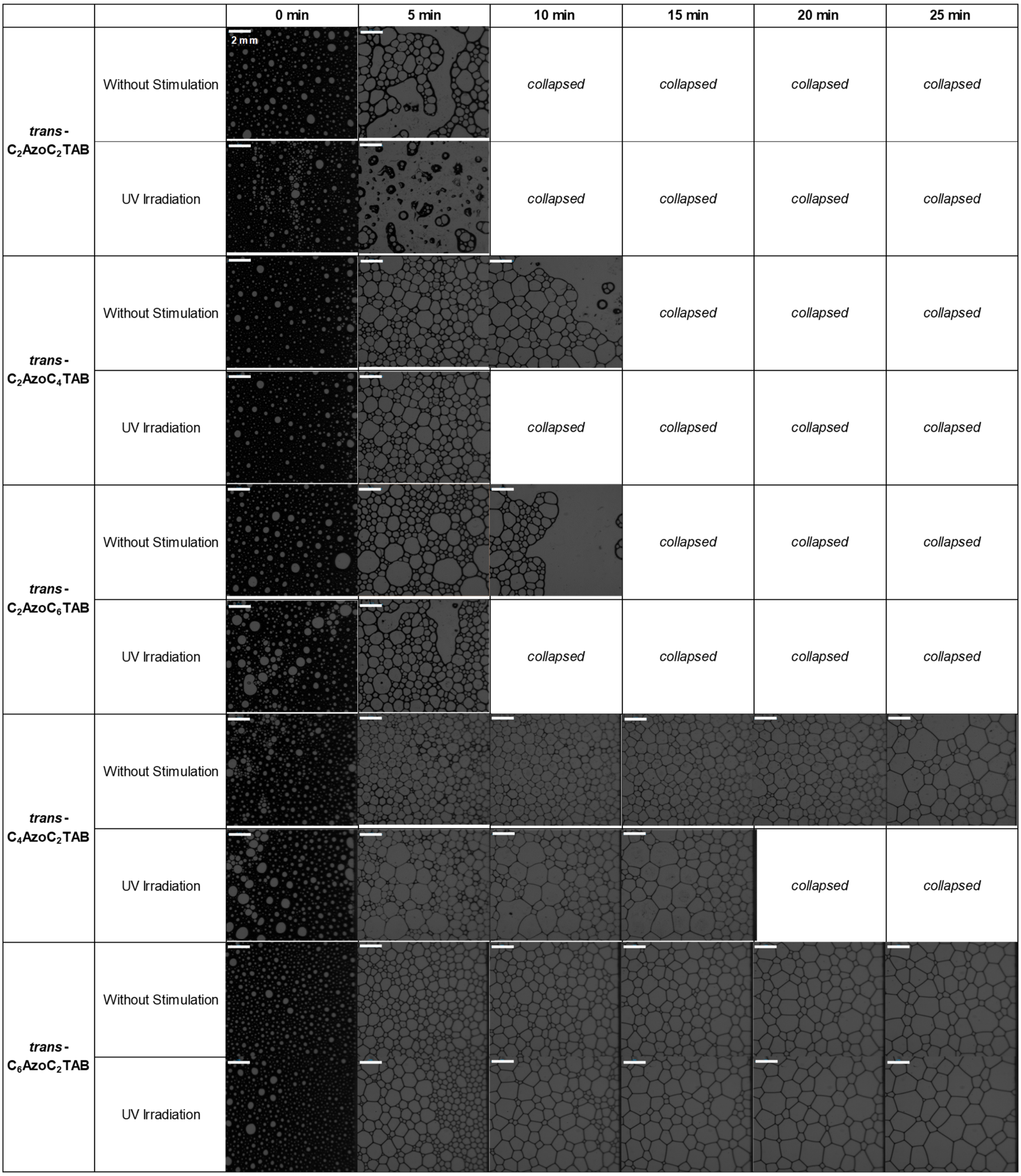

Figure S6. Time-dependent images of the foam structure for C_mAzoC_nTAB solution at $C_{\Delta\gamma\max}$.

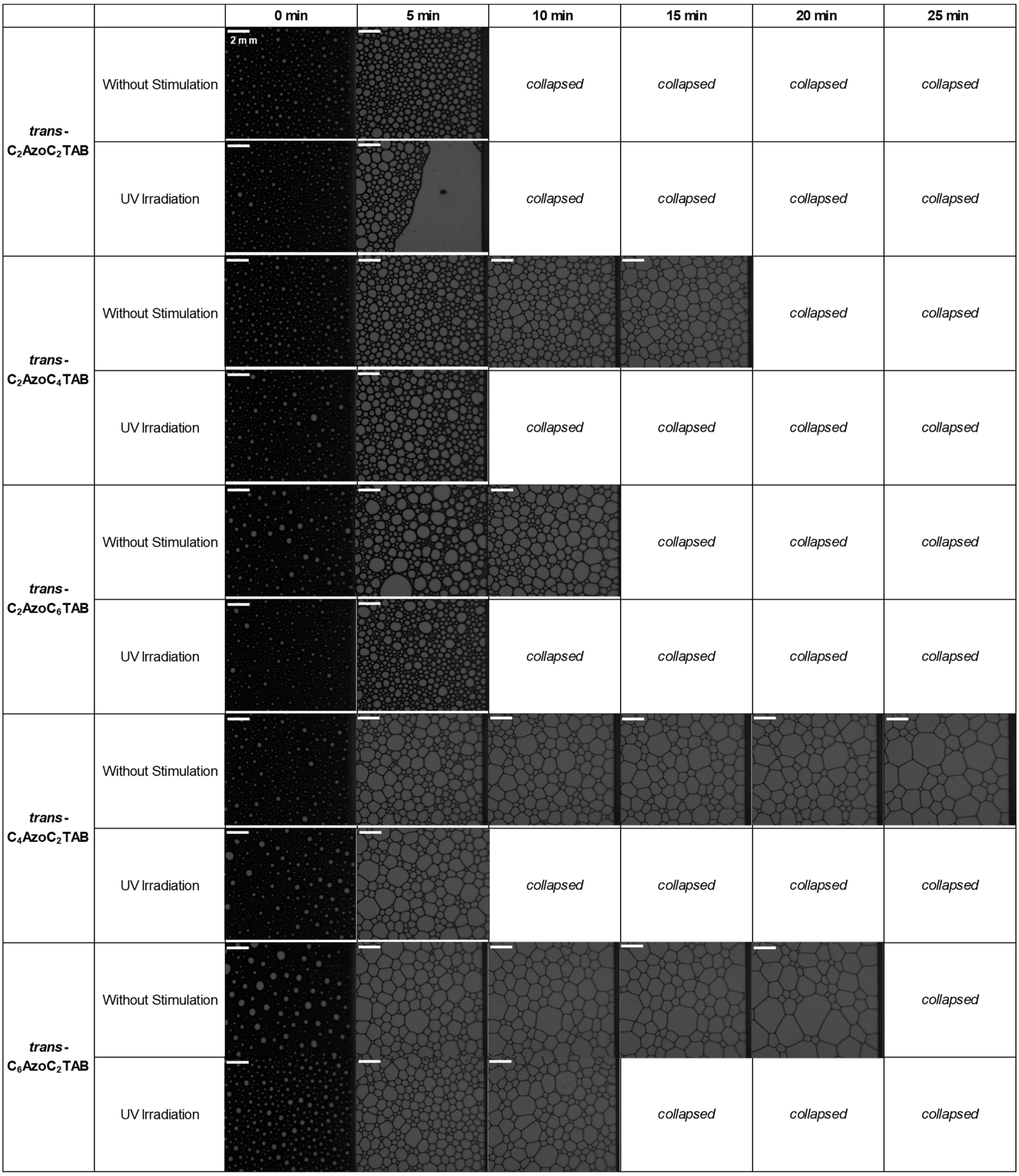

Figure S7. Time-dependent images of the foam structure for C_mAzoC$_n$TAB solution at 10 mmol dm^{-3}.

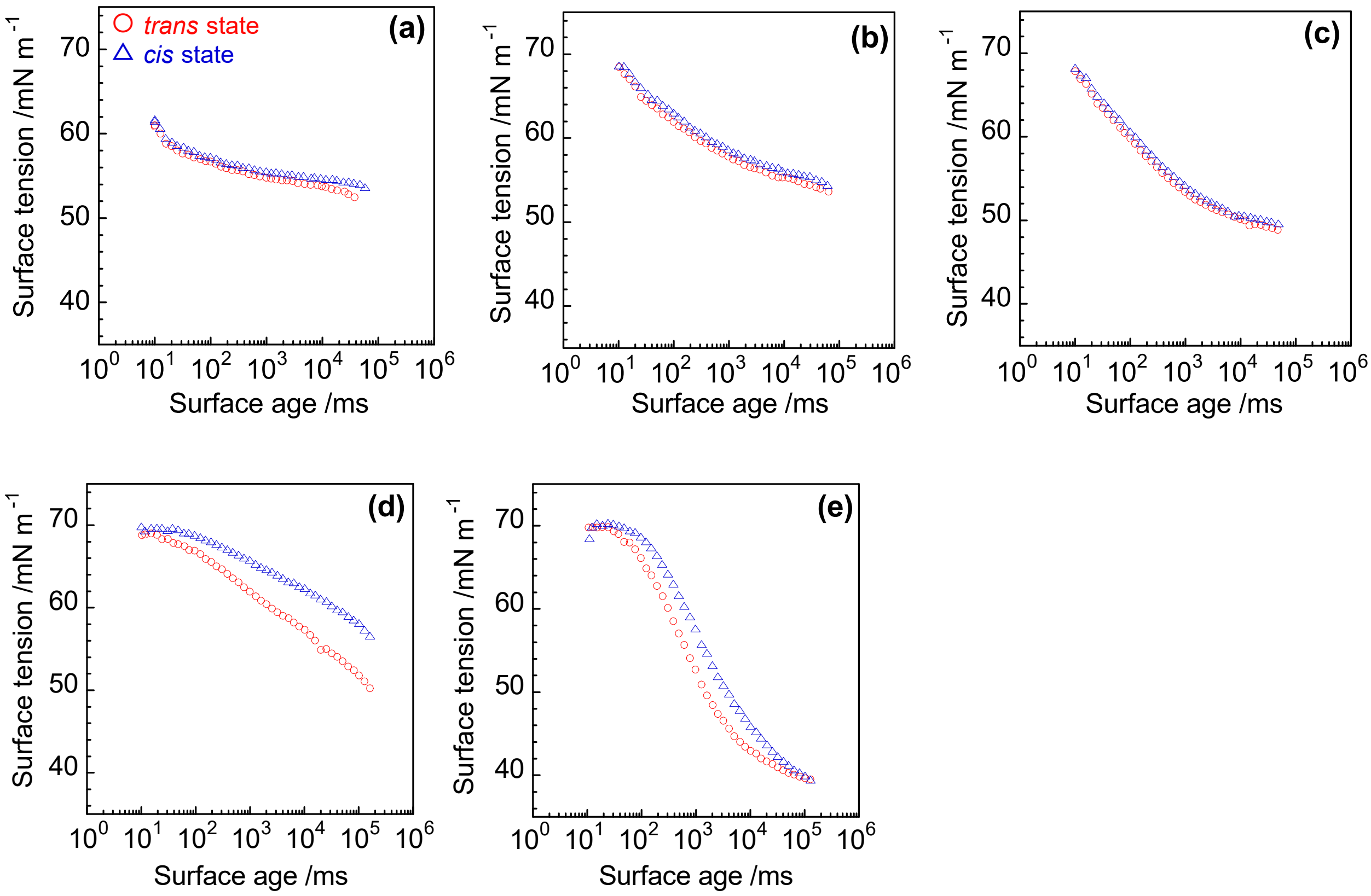

Figure S8. Dynamic surface-tension profiles of the *trans* and *cis* states of C_mAzoC_nTAB surfactants measured at their respective $C_{\Delta\gamma\max}$ concentrations: (a) C_2AzoC_2TAB, (b) C_2AzoC_4TAB, (c) C_2AzoC_6TAB, (d) C_4AzoC_2TAB, and (e) C_6AzoC_2TAB.
